# Towards Multi-Analyte Detection with Field-Effect Capacitors Modified with *Tobacco Mosaic Virus* Bioparticles as Enzyme Nanocarriers

**DOI:** 10.3390/bios12010043

**Published:** 2022-01-14

**Authors:** Melanie Welden, Arshak Poghossian, Farnoosh Vahidpour, Tim Wendlandt, Michael Keusgen, Christina Wege, Michael J. Schöning

**Affiliations:** 1 Institute of Nano- and Biotechnologies, Aachen University of Applied Sciences, 52428 Jülich, Germany; m.welden@fh-aachen.de (M.W.); vahidpour@fh-aachen.de (F.V.); 2 Institute of Pharmaceutical Chemistry, Philipps University Marburg, 35032 Marburg, Germany; michael.keusgen@staff.uni-marburg.de; 3 MicroNanoBio, 40479 Düsseldorf, Germany; a.poghossian@gmx.de; 4 Institute of Biomaterials and Biomolecular Systems, University of Stuttgart, 70569 Stuttgart, Germany; tim.wendlandt@bio.uni-stuttgart.de (T.W.); christina.wege@bio.uni-stuttgart.de (C.W.); 5 Institute of Biological Information Processing (IBI-3), Forschungszentrum Jülich GmbH, 52425 Jülich, Germany

**Keywords:** *tobacco mosaic virus* (TMV), capacitive field-effect sensor, bi-enzyme biosensor, enzyme-logic gate, urease, penicillinase

## Abstract

Utilizing an appropriate enzyme immobilization strategy is crucial for designing enzyme-based biosensors. Plant virus-like particles represent ideal nanoscaffolds for an extremely dense and precise immobilization of enzymes, due to their regular shape, high surface-to-volume ratio and high density of surface binding sites. In the present work, *tobacco mosaic virus* (TMV) particles were applied for the co-immobilization of penicillinase and urease onto the gate surface of a field-effect electrolyte-insulator-semiconductor capacitor (EISCAP) with a p-Si-SiO_2_-Ta_2_O_5_ layer structure for the sequential detection of penicillin and urea. The TMV-assisted bi-enzyme EISCAP biosensor exhibited a high urea and penicillin sensitivity of 54 and 85 mV/dec, respectively, in the concentration range of 0.1–3 mM. For comparison, the characteristics of single-enzyme EISCAP biosensors modified with TMV particles immobilized with either penicillinase or urease were also investigated. The surface morphology of the TMV-modified Ta_2_O_5_-gate was analyzed by scanning electron microscopy. Additionally, the bi-enzyme EISCAP was applied to mimic an **XOR** (**Exclusive OR**) enzyme logic gate.

## 1. Introduction

The field effect in an electrolyte-insulator-semiconductor (EIS) system offers a universal transducer principle for designing many kinds of chemical sensors and biosensors (see e.g., recent reviews [1,2,3,4,5,6,7,8,9]). EIS capacitors (EISCAP) are considered as the simplest type of such field-effect sensors [10]. They have been implemented for detecting various biochemical species such as ions [11,12,13,14], charged molecules (DNA (deoxyribonucleic acid) [15,16,17], protein biomarkers [18,19,20,21,22], polyelectrolytes [23,24]), virus-like particles [25,26], ligand-stabilized nanoparticles [27,28], etc. In addition, numerous enzyme-modified EISCAP biosensors were constructed for the detection of various analytes such as glucose [29,30,31], urea [30,31,32], creatinine [33], penicillin [31,34], formaldehyde [35], triglycerides [36], and acetoin [37]. The operation mechanism of these biosensors is based on the detection of local pH changes resulting from the catalytic reaction of the immobilized enzyme on the sensor surface with its specific substrate [10]. Moreover, the ability of EISCAP sensors for multi-analyte detection using a single EISCAP chip or an array of EISCAPs has been demonstrated [31,38,39,40,41,42,43,44].

Generally, the analytical characteristics of enzyme-based biosensors are strongly affected by the enzyme immobilization method [45,46]. Therefore, the choice of an appropriate enzyme immobilization strategy is a key factor for designing the biorecognition part of biosensors, including EISCAPs. The immobilization method must provide a high enzyme load on the biosensor surface, a good accessibility of the active sites for the target analytes, as well as to retain the structure, function, and catalytic activity of the enzyme [45,46]. Intensive efforts have been made during the last decade to develop novel immobilization techniques to improve the biosensor performance: the use of nanomaterials (e.g., metal, oxide or organic nanoparticles, nanowires, carbon nanotubes, magnetic beads) as nanoscale scaffolds for the immobilization of receptor molecules [47,48,49,50] as well as the incorporation of enzymes within nanoscale structures (e.g., alginate gels [51], polyelectrolyte/enzyme [52] or carbon nanotube/enzyme multilayers [53]). More recently, due to their regular shape, high surface-to-volume ratio, and extremely high density of surface docking sites, biological nanoscaffolds such as plant virus-like particles have increasingly been used for the precisely positioned immobilization of receptors on different transducers for biosensing purposes [54,55,56]. In contrast to various chemically synthesized nanoparticles, which are typically polydisperse with a more randomized and unreproducible distribution of particle size and density of shell molecules, many types of virus particles are monodisperse with regard to uniform morphologies and specific dimensions.

The *tobacco mosaic virus* (TMV) is one of the most comprehensively investigated plant viruses. Native full-length TMV particles are 300 nm long, nanotube-like nucleoprotein complexes with inner and outer diameters of 4 and 18 nm, respectively [56,57]. TMV particles exhibit excellent chemical and physical stability: in solutions, they can withstand 90 °C [58] and pH values between pH 3 and pH 9 [57]. The outer surface of each TMV nanotube holds thousands of docking sites accessible on about 2130 identical, helically arranged coat protein (CP) subunits, capable in the coupling of functional molecules. Therefore, TMV particles have often been utilized as biological nanoscaffolds for an extremely dense and precisely controlled immobilization of biorecognition molecules (receptors), including enzymes [55,56,59]. Moreover, TMV particles functionalized with bioreceptors can be simply combined with different transducer structures for chemical and biological sensing. For example, TMV particles were applied to assist the detection of the explosive agent trinitrotoluene [60], volatile organic compounds [61], and antigen-antibody binding [62,63]. The authors introduced amperometric glucose [64] as well as colorimetric and field-effect penicillin biosensors [65,66] by using TMV particles as nanoscaffolds for the immobilization of glucose oxidase and penicillinase, respectively. TMV particles as receptor nanocarriers usually enable increased receptor densities per sensor area, an enhanced mass transport of the target molecules to the TMV surface in comparison to planar surfaces, and a favorable orientation of the receptor molecules for an increased receptor-substrate interaction; all these factors enhance the overall biosensor performance [56,64].

In this work, the ability of TMV-assisted enzyme-based EISCAPs for multi-analyte detection was demonstrated. Penicillinase and urease were co-immobilized onto TMV surfaces for the sequential detection of penicillin and urea using the same p-Si-SiO_2_-Ta_2_O_5_ field-effect sensor. These two enzyme/substrate systems (i.e., penicillinase/penicillin and urease/urea) represent typical model experiments inducing counter-rotating pH shifts on the sensor surface: a) generating hydrogen ions (penicillinase/penicillin → pH decrease) or b) consuming hydrogen ions (urease/urea → pH increase). For comparison, TMV-modified EISCAPs immobilized with either penicillinase or urease (no co-immobilization) were also studied. Finally, in a proof-of-concept experiment, the newly developed TMV-assisted bi-enzyme EISCAP biosensor was applied to mimic an XOR (Exclusive OR) enzyme logic gate.

## 2. Materials and Methods

### 2.1. Preparation of Biotinylated Tobacco Mosaic Virus Particles

A TMV variant (TMV_Cys_) containing a S3C mutation close to the N-terminus of every CP was employed as a viral enzyme nanocarrier with a spacing of 2.5 to 3.5 nm of coupling sites on their outer protein coat [67]. This mutant exposed 2130 cysteine residues on each TMV particle presenting surficial sulfhydryl groups. TMV_Cys_ extracted from infected *Nicotiana tabacum* leaves were equipped with PEG_11_-biotin moieties via maleimide-sulfhydryl-coupling (EZ-Link® Maleimide-PEG_11_-Biotin, Thermo Scientific, Rockford, IL, USA) according to [56]. Briefly, a maleimide-PEG_11_-biotin linker was incubated with TMV_Cys_ particles in a molar ratio of 3:1 (linker/CP) for 3 h at 26 °C under agitation. Unbound linker molecules were removed by centrifugal ultrafiltration (Amicon Ultra, 30 kDa molecular weight cut-off, Merck-Millipore, Darmstadt, Germany) in five consecutive washing steps with 10 mM of sodium-potassium-phosphate (SPP) buffer (pH 7.0). The resulting biotinylated TMV_Cys_ (TMV_Cys_/_Bio_) particles were resuspended and stored in this buffer at 4 °C. The particles were analyzed by sodium dodecyl sulphate–polyacrylamide gel electrophoresis and colloidal Coomassie Brilliant Blue G-250 staining [68]. Densitometric comparison of the signals corresponding to the biotinylated and the non-modified form of the CP confirmed biotinylation of >90 % of the CPs.

### 2.2. Preparation of Streptavidin-Enzyme Conjugates

Penicillinase from *Bacillus cereus* (1500–3000 Units/mg protein, Sigma Aldrich, Darmstadt, Germany) and urease from *Canavalia ensiformis* (75,265 Units/g solid, from Jack bean, Sigma Aldrich, Germany) were applied as the model enzymes. In order to immobilize them on the biotinylated TMV particles, they were conjugated with streptavidin (SA), allowing strong attachment via SA-biotin high affinity binding. The streptavidin conjugation was performed by utilizing a commercial conjugation kit (LYNX Streptavidin rapid conjugation kit, Bio-Rad, Great Britain). The conjugation of penicillinase was performed as described in [65], using a molar ratio between penicillinase and SA of 1:25 in the reaction mixture. For the urease conjugation, the same protocol was applied with a molar ratio of 1:10. The streptavidin-conjugated enzymes (SA-enzymes) were stored in stock solutions (10 mM phosphate buffered saline (PBS)) with a concentration of 600 Units/mL (SA-penicillinase) and of 2000 Units/mL (SA-urease) at 4 °C. For the fabrication of the bi-enzyme biosensors, both enzyme solutions were mixed in a ratio of 1:1, resulting in an enzyme cocktail containing 300 Units/mL SA-penicillinase and 1000 Units/mL SA-urease.

### 2.3. Modification of EISCAP Sensors with TMV Particles and Coupling of SA-Enzyme Conjugates

**Figure 1:**
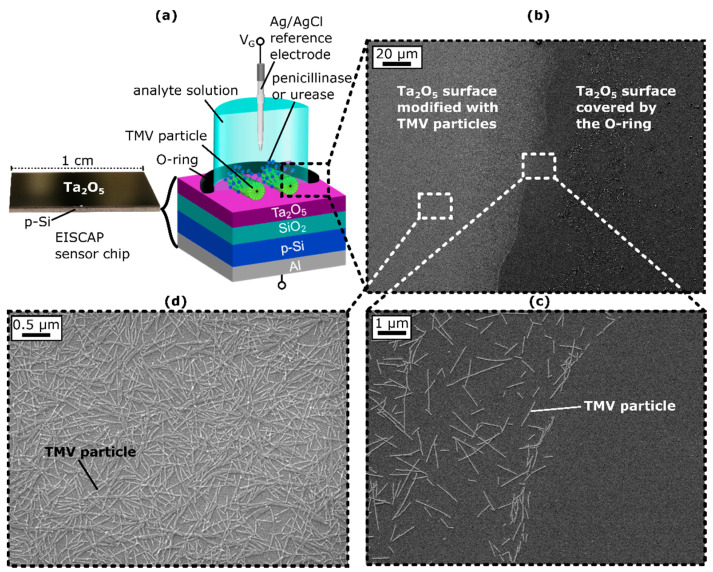
(**a**) Photo of the EISCAP sensor chip (left) and schematic layer structure (right) of the TMV-assisted Al/p-Si/SiO_2_/Ta_2_O_5_-EISCAP sensor modified with penicillinase and/or urease, mounted in a measurement cell and sealed by an O-ring. (**b**) Scanning electron microscopic (SEM) image of the Ta_2_O_5_-gate surface showing the distinguished areas where the Ta_2_O_5_ surface is covered by the O-ring preventing TMV adsorption (right) and the area inside the O-ring, where the Ta_2_O_5_ is modified with TMV particles (left). (**c**) Magnification of the border line between TMV particle-modified and bare Ta_2_O_5_ surface. (**d**) Zoomed out image of the TMV particle-modified surface area. *V*_G_: gate voltage.

In this study, EISCAPs with an Al/p-Si/SiO_2_ (30 nm)/Ta_2_O_5_ (60 nm) layered structure were used as the sensor platform, as schematically represented in Figure 1a. Details of the fabrication process were described in [25]. Prior to their modification with the TMV particles and enzymes, the sensors were cleaned in an ultrasonic bath for 5 min each with acetone, isopropanol, ethanol, and deionized water, and then installed into a homemade measurement cell. In the measurement cell, the sensor chip was sealed by an O-ring, with 0.5 cm² of the sensor surface in contact with the electrolyte (see Figure 1a). A 50 µL TMV solution (0.1 mg/mL) was incubated for one hour at room temperature (RT) on this exposed Ta_2_O_5_ surface to allow TMV adsorption. Subsequently, not-adsorbed TMV particles were washed away with 10 mM PBS buffer. Afterwards, 50 µL of the particular SA-enzyme solution (single enzyme EISCAP: 50 µL SA-penicillinase or 50 µL SA-urease solution; bi-enzyme EISCAP: 50 µL SA-penicillinase + SA-urease solution) were incubated for two hours at RT. The sensor surface was then flushed three times with 0.33 mM PBS and conditioned in 0.33 mM PBS buffer solution for at least one hour, before the electrochemical measurements were started. When the EISCAPs were not in use, they were stored in 0.33 mM PBS buffer at 4 °C.

### 2.4. Electrochemical Characterization of TMV-Modified EISCAP Biosensors

For electrochemical characterization of the TMV-assisted EISCAPs, an Ag/AgCl reference electrode (filled with 3 M KCl, Metrohm, Filderstadt, Germany) was immersed in the buffer solution and connected to an impedance analyzer (Zahner Zennium, Zahner Elektrik, Kronach, Germany). Furthermore, the Al rear-side contact was electrically connected to the impedance analyzer. All measurements were performed in a measurement buffer (0.33 mM PBS buffer) with varying concentrations of penicillin G (Sigma Aldrich, Darmstadt, Germany) and/or urea (GE Healthcare Bio-Sciences AB, Uppsala, Sweden) at RT. In order to avoid signal interferences, the set-up (except of the impedance analyzer) was integrated in a dark Faraday cage. As a first step, leakage-current measurements were carried out in a measurement solution without penicillin and urea. Therefore, gate voltages from –3 V to +3 V were applied with 100 mV steps between the reference electrode and the Al rear-side contact. These measurements served as a quality control of the insulator layer, where only sensors exhibiting a leakage current < 10 nA were selected for further electrochemical measurements. In the next step, capacitance–voltage (*C–V*) measurements were performed in the measurement buffer (by applying a gate voltage between –2 V and +2 V with 100 mV steps) to check the correct functioning of the field-effect EISCAPs. In order to measure the capacitance of the EISCAP, a small AC (alternating current) voltage of 20 mV with a frequency of 120 Hz was superimposed. For the following constant-capacitance (ConCap) measurements, a working point was set in the depletion region of the *C–V* curve at about 60% of the maximum capacitance. The ConCap mode offered the time-dependent detection of surface-charge (potential) changes induced by, e.g., local pH changes at the sensor surface. During the ConCap mode, the capacitance of the sensor structure in the working point was kept constant by a control loop: Changes of the surface potential, e.g., induced by the enzymatic conversion of penicillin or urea, were compensated by applying an opposed voltage at the reference electrode. These voltage changes were recorded over time, allowing for the dynamic detection of changes in the penicillin and urea concentrations. A detailed description of the *C–V* and ConCap-measurement mode was provided in [23]. The ConCap measurements were conducted in a measurement buffer as well as in penicillin and urea solutions with concentrations between 0.1 and 5 mM for single-enzyme sensors and between 0.1 mM and 3 mM for bi-enzyme sensors. The penicillin and urea were purchased from Sigma Aldrich (Darmstadt, Germany) and GE Healthcare Bio-Sciences AB (Uppsala, Sweden), respectively.

### 2.5. Characterization of Surface Morphology by SEM

To control and characterize the TMV adsorption on the Ta_2_O_5_-gate surface, SEM images of the TMV-modified sensor chip were taken using a JEOL JSM-7800F Schottky field-emission microscope (JEOL GmbH, Freising, Germany). For this purpose, the sensors were mounted out of the measurement chamber, rinsed with deionized water, and dried with nitrogen to remove the salt residues of the buffer solution. Subsequently, an approximately 5 nm thick platinum-palladium layer was sputtered onto the sensor surface to provide conductivity and prevent the additional charging of the sensor surface.

## 3. Results and Discussion

### 3.1. SEM Images of TMV-Modified EISCAPs

To ensure that the TMV particles had adsorbed to the Ta_2_O_5_-gate surface within the O-ring and thus could act as nanocarriers for enzyme attachment, SEM images of the sensor surface were obtained after TMV loading. Figure 1b–d shows exemplary SEM images of the chip surface with a clear boundary between the TMV-modified region and the area sealed by the O-ring (see Figure 1c). Within the O-ring, TMV particles were homogeneously distributed on the Ta_2_O_5_ surface, with some virus particles present as lateral or head-to-tail aggregates (Figure 1d), as is typical for TMV particles [25,26,66]. The TMV particles were adsorbed in a high density of about 6.3 × 10^9^ particles/cm^2^ on the Ta_2_O_5_ surface, which was slightly higher than was reported in previous works [25,26,66] and revealed that the TMVs had been successfully attached to the Ta_2_O_5_ surface where they were available for enzyme immobilization.

### 3.2. TMV-Assisted Single-Enzyme EISCAPs

In order to study the sensor performance of TMV-assisted single enzyme EISCAPs, penicillin and urea biosensors with penicillinase and urease, respectively, were fabricated separately. Penicillinase catalyzes the conversion of penicillin to penicilloic acid, whereby H^+^ ions are produced, leading to a local pH decrease [69]. In contrast, during the hydrolysis reaction of urea catalyzed by the urease, OH^–^ ions are produced (or H^+^ ions are consumed), resulting in a local pH increase [70,71]. Changes in pH at the EISCAP surface cause the surface to become more positively charged (in the case of a pH decrease) or more negatively charged (in the case of a pH increase). This surface-charge change influences the width of the space-charge region in the semiconductor layer and, thus, the total capacitance of the sensor structure. Choosing these two enzymes enabled investigating whether EISCAPs modified with TMV particles as enzyme nanocarriers were suitable for enzymatic reactions involving both acidification and alkalization. The direction of the EISCAP-signal changes would directly correlate with these two kinds of enzymatic reactions, generating (penicillinase/penicillin) or consuming (urease/urea) hydrogen ions.

#### 3.2.1. Penicillin Biosensor

**Figure 2:**
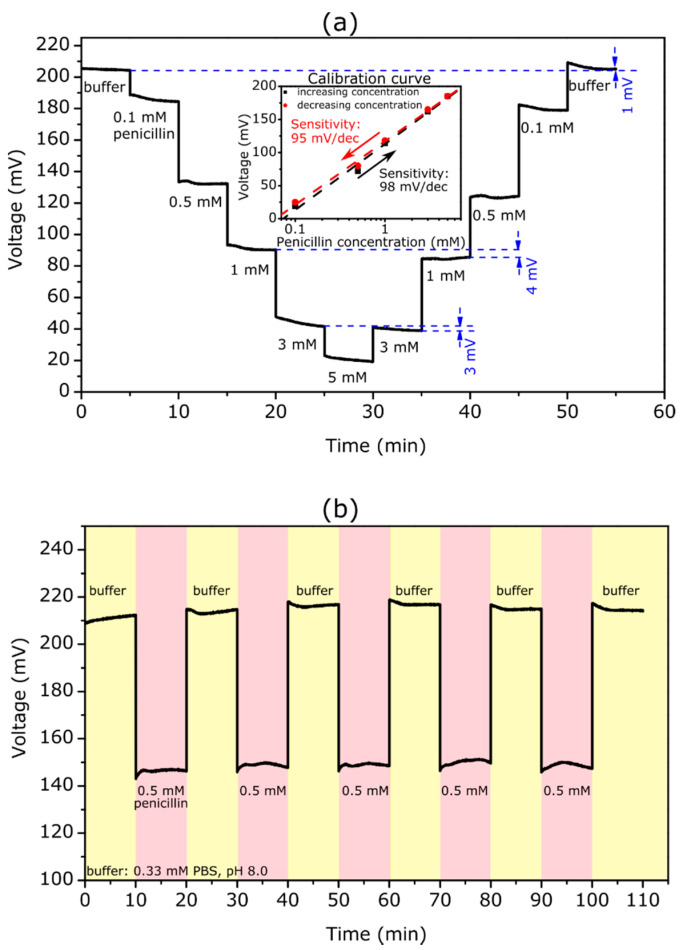
(**a**) ConCap curve of a TMV/SA-penicillinase-modified EISCAP recorded in 0.33 mM PBS buffer (pH 8.0) with different penicillin concentrations between 0.1 and 5 mM. The inset figure presents the resulting calibration curves with a penicillin sensitivity of about 98 and 95 mV/dec for the increasing (black) and decreasing (red) concentration series of measurements, respectively. (**b**) Reproducibility of the TMV-assisted EISCAP penicillin biosensor: the ConCap signal was repeatedly measured in buffer and in 0.5 mM penicillin solution in alternating order.

Figure 2a shows a ConCap curve of the TMV/SA-penicillinase modified EISCAP biosensor in the loop of penicillin concentrations of 0.1, 0.5, 1, 3, 5, 3, 1, 0.5, and 0.1 mM. The measurements were carried out in 0.33 mM PBS at pH 8.0, which corresponds to the pH optimum of the penicillinase [72]. For each penicillin concentration, the ConCap signal was recorded for approximately 5 min.

As expected, with increasing penicillin concentration, the recorded signal shifted towards less positive voltages. By increasing the penicillin concentration, more H^+^ ions were generated and the local pH value at the sensor surface became lower: The produced H^+^ ions protonate the hydroxyl groups on the Ta_2_O_5_ surface making it more positively charged. Consequently, the total capacitance of the EISCAP was decreased. To keep the total capacitance of the sensor constant, the applied voltage at the reference electrode should become more negative (or less positive), which is visible as a shift in the ConCap signal. Conversely, with decreasing penicillin concentration, the ConCap signal shifted in the opposite direction. The clear steps at different penicillin concentrations also underlined a fast response time of the TMV-based penicillin biosensor. 

The measurement curve revealed that the sensor had a low hysteresis of 1 and 3 mV in the buffer without penicillin and in the 3 mM penicillin solution, respectively. However, it was observed that hysteresis is somewhat higher at lower penicillin concentrations with a maximum value of 8 mV at 0.5 mM. Generally, hysteresis of pH-sensitive field-effect devices is interpreted as slow response due to the slow buried sites underneath the gate-insulator surface (see e.g., [73,74]). It was reported that the hysteresis increases with an increasing pH-loop time, and in acid solutions it is smaller than in alkaline solutions [73,74] In addition, the hysteresis width may be affected by the background long-term drift, slow states at the Si-SiO_2_ interface, as well as by the possible alteration of the enzyme activity due to local pH changes. The experiments performed in this study did not allow us to provide a clear explanation for the analyte-concentration dependence of the hysteresis width. Therefore, additional in-depth studies are needed to quantify this phenomenon.

The inset figure indicated the calibration plots for upward and downward penicillin-concentration loops evaluated from the ConCap response. The TMV-assisted EISCAP biosensor exhibited a high penicillin sensitivity of about 98 and 95 mV/dec for the increasing and decreasing concentration series of measurements, respectively. The small difference in penicillin sensitivities of 3 mV/dec observed for upward and downward concentration loops could be attributed to the hysteresis effect. The obtained sensitivity values were comparable to our previous work with TMV-based penicillin biosensors [66] and were higher than sensitivity values obtained for EISCAPs with adsorptively immobilized penicillinase (68.7 mV/dec) [31].

One of the important operation characteristics of biosensors is the reproducibility of the sensor response. To demonstrate the reproducibility of the TMV-assisted EISCAP penicillin biosensor, the ConCap signal was repeatedly measured in buffer (six times) and in a 0.5 mM penicillin solution (five times) in alternating order. The results of these experiments are shown in Figure 2b. The mean signal was 67 ± 1 mV, which underlines the high reproducibility of the developed penicillin biosensor.

#### 3.2.2. Urea Biosensor

**Figure 3:**
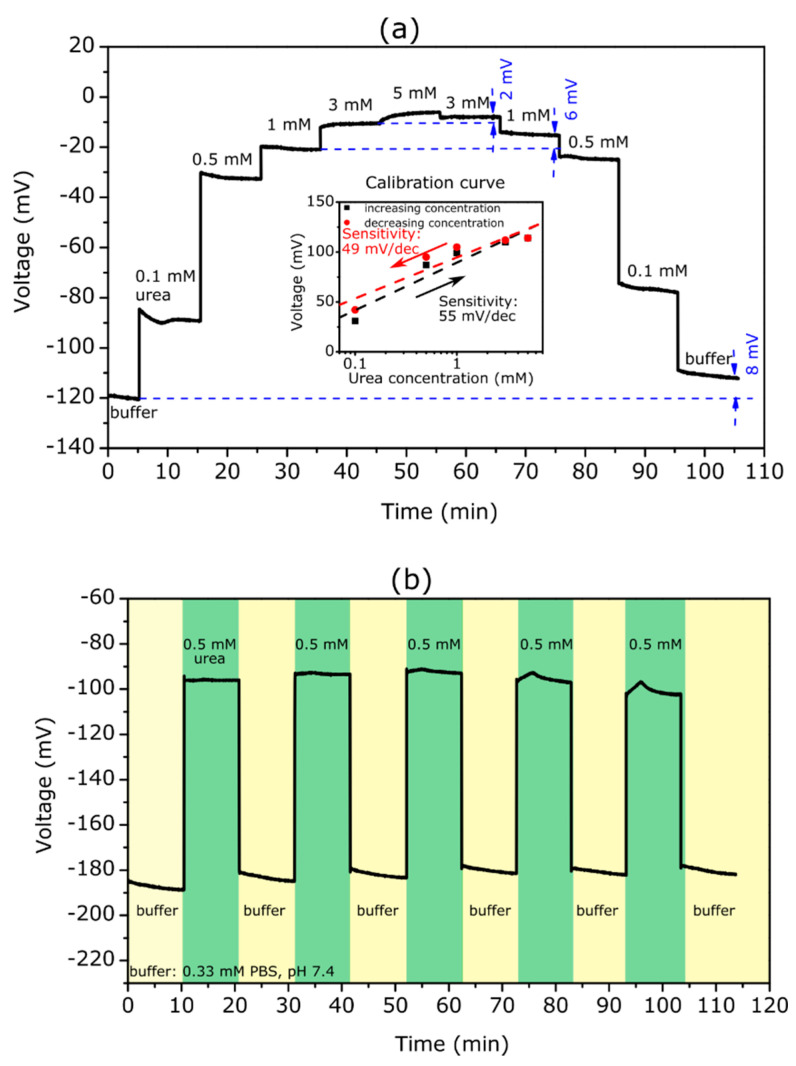
(**a**) ConCap curve of a TMV/SA-urease-modified EISCAP recorded in 0.33 mM PBS buffer (pH 7.4) with different urea concentrations between 0.1 and 5 mM. The inset figure presents the resulting calibration curves with a urea sensitivity of about 55 and 49 mV/dec for increasing (black) and decreasing (red) concentration series of measurements, respectively. (**b**) Reproducibility of the TMV-assisted EISCAP urea biosensor: the ConCap signal was repeatedly measured in buffer solution and in 0.5 mM urea solution in alternating order.

Figure 3a depicts a ConCap curve of an EISCAP modified with TMV/SA-urease, which was recorded in 0.33 mM PBS at pH 7.4, specified by the supplier as the optimum pH for urease [75].

Here, the ConCap signal behaved in the opposite way to that of the penicillin biosensor: with an increasing urea concentration from 0.1 to 5 mM, the measurement signal rose in the direction of the more positive (or less negative) voltages. The observed signal behavior could be explained as follows: an increase of the urea concentration resulted in a rise of the local pH value and deprotonation of the hydroxyl groups on the Ta_2_O_5_ surface, making it more negatively charged, whereby the total capacitance of the EISCAP increased. To keep the overall capacitance of the EISCAP sensor constant, the applied voltage at the reference electrode must be more positive (or less negative), which appeared as a signal shift in the ConCap curve.

Again, as with the penicillin measurements, clearly delineated signal levels could be seen at different urea concentrations with a low hysteresis of 2 mV for 3 mM urea. However, the hysteresis width increased with a decreasing urea concentration and amounts of 11 mV at 0.1 mM. In contrast to the penicillin measurements, a relatively large hysteresis of 8 mV was also recorded in the buffer solution. Additionally, at low urea concentrations, it took longer before a stable sensor signal was achieved. From the ConCap response at different urea concentrations, the calibration curves for the upward and downward concentration loops were evaluated, which are depicted in the inset figure. The TMV-assisted urea biosensor reveals a high urea sensitivity of 55 and 49 mV/dec for the increasing and decreasing concentration series of measurements. The difference in urea sensitivities for the upward and downward concentration loops was larger (6 mV/dec) than that of the TMV-assisted penicillin biosensor. In other studies with urea sensors based on EISCAPs, urea sensitivities of 16 mV/dec (1 to 100 mM) by using a layer-by-layer nanofilm of ZnO nanocrystals and carbon nanotubes [76], 32 mV/dec by the immobilization of urease on magnetic particles [71], and 40.5 mV/dec (1 to 25 mM) in the case of nano-spotted urease [31] were achieved. Thus, the EISCAP modified with TMV/SA-urease offers a higher sensitivity and the ability of detecting low urea concentrations.

The results of the reproducibility experiments are shown in Figure 3b. Like the penicillin biosensor, the ConCap signal of the TMV-assisted EISCAP urea biosensor was repeatedly measured in buffer (six times) and in a 0.5 mM urea solution (five times) in alternating order. The urea biosensor signal was reproducible with a mean signal of (88 ± 5) mV.

### 3.3. TMV-Assisted Bi-Enzyme EISCAP Biosensor

**Figure 4:**
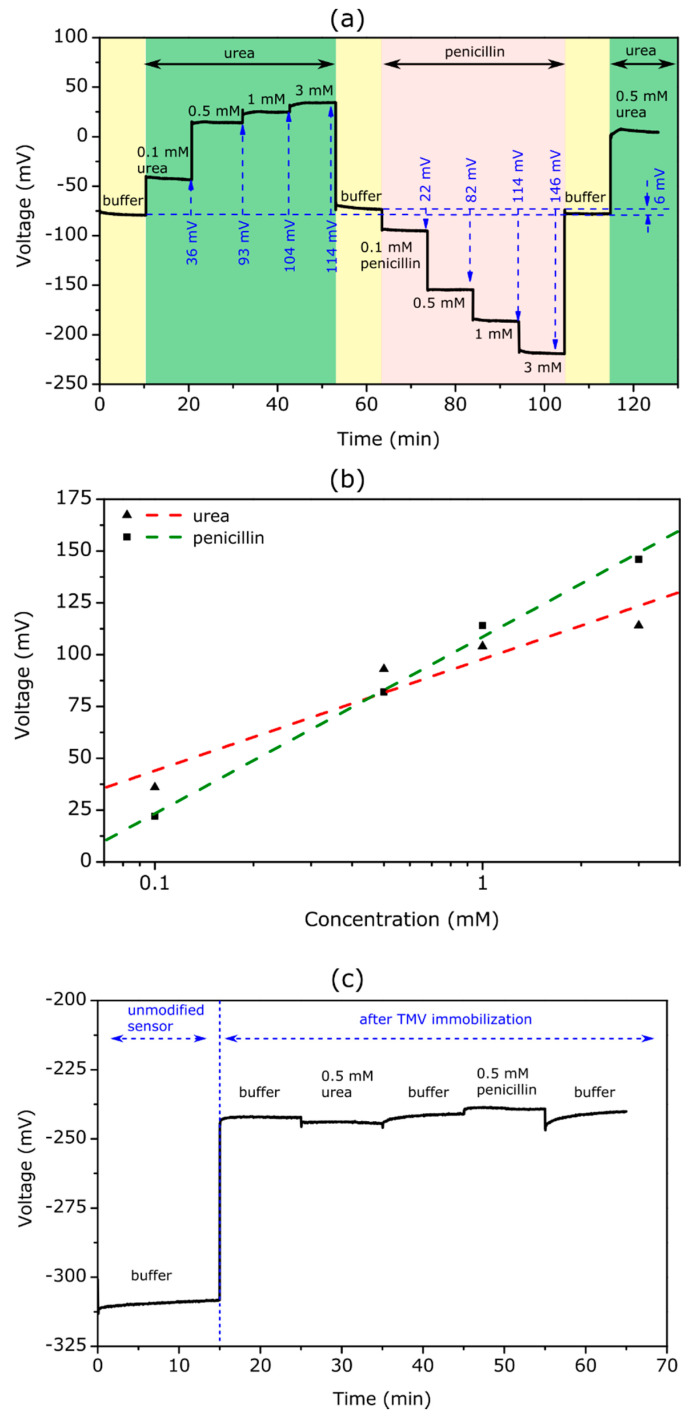
(**a**) ConCap curve of the TMV-assisted bi-enzyme EISCAP biosensor recorded in buffer and at different concentrations of urea and penicillin. (**b**) Calibration curves for urea and penicillin evaluated from the ConCap response in (**a**). (**c**) ConCap curve recorded with an EISCAP sensor before and after TMV loading (without enzymes) in 0.33 mM PBS (pH 7.4) and in 0.33 mM PBS (pH 7.4) with 0.5 mM urea and 0.5 mM penicillin, respectively.

The TMV-assisted bi-enzyme EISCAP, where both enzymes had been co-immobilized on the same sensor chip, was applied for the serial detection of urea and penicillin for the first time. Figure 4a depicts the ConCap response at different urea and penicillin concentrations ranging from 0.1 to 3 mM.

The measurements were performed in the following order: First, the ConCap response was recorded in the buffer solution (0.33 mM PBS, pH 7.4) to obtain the baseline signal for the sensor, followed by measurements in urea solutions with different concentrations. Second, the sensor signal was recorded again in the buffer solution to obtain the baseline signal for the subsequent penicillin measurements with varying penicillin concentrations. Finally, the ConCap signal was recorded again in the buffer and urea solution. Such sequential arrangements of experiments are useful to examine the recoverability of the sensor signal as well as to identify possible hysteresis and drift effects.

As expected, the bi-enzyme sensor showed distinct signal steps towards more positive voltages with an increasing urea concentration and towards more negative voltages with an increasing penicillin concentration. For example, at a urea and penicillin concentration of 3 mM, the signal shift reached approximately 114 and 146 mV, respectively. Taking into account a pH sensitivity of 56 mV/pH for the Ta_2_O_5_-gate EISCAPs studied in this work, the local pH value on the TMV/SA-enzyme modified EISCAP surface was estimated to be pH = 9.4 or pH = 4.8 at a urea or penicillin concentration of 3 mM, respectively. This indicated the stability of the TMV/SA-enzyme system on the EISCAP surface over a wide pH range.

In Figure 4b, the evaluated calibration curves of the TMV-assisted bi-enzyme EISCAP for urea and penicillin are illustrated. The biosensor exhibited a urea sensitivity of 54 mV/dec, which was nearly similar to that of the single-enzyme biosensor. The penicillin sensitivity of the bi-enzyme EISCAP amounted to 85 mV/dec, which was slightly lower than that achieved with the single-enzyme EISCAP (see Section 3.2.1). The results indicated that both enzymes maintained their activity after the co-immobilization.

In general, a possible non-specific adsorption of analyte molecules onto the gate surface of the field-effect device may induce an unwanted background signal, and, thus, may reduce the signal-to-noise ratio of the sensor signal [1,4,10]. In our case, such non-specific adsorption may occur either on the TMV particles or on TMV-free areas of the gate surface of the EISCAP. Therefore, we studied the signal behavior of the TMV-modified (but enzyme-free) EISCAP in the urea and penicillin solutions. Figure 4c depicts the ConCap signal of the EISCAP recorded in 0.33 mM PBS (pH 7.4) buffer before (unmodified sensor) and after the loading of TMV particles, followed by measurements in buffer containing 0.5 mM urea or penicillin. As TMV particles are negatively charged [26], the sensor signal shifted to more positive voltages after their loading onto the Ta_2_O_5_ surface. However, upon subsequent measurements in analyte solutions, the signal remained almost constant, indicating that the existence of urea or penicillin molecules in the solution had practically no impact on the EISCAP sensor signal.

### 3.4. XOR Logic Gate Using TMV-Assisted Bi-Enzyme Biosensor 

In the past, intensive research was performed in the field of enzyme-based logic gates, which mimic the operation of electronic logic gates (see e.g., [77,78]). In enzyme logic gates, Boolean logic operations are activated by specific molecular inputs via enzymatic reactions. An interfacing of enzyme logic gates with electronic transducers was considered a very promising approach for designing digital biosensors that could provide qualitative evidence (in a YES/NO format) concerning the presence or absence of a specific analyte in the sample [79,80,81]. In previous works, we demonstrated the successful integration of enzyme logic gates with pH-sensitive EISCAPs as **AND**-Reset, **OR**-Reset, **CNOT** (**controlled NOT**) and **XOR** gates [82,83,84,85]. In these logic gate devices, enzymes were immobilized onto the biosensor surface by means of physical entrapment within a membrane or through physical adsorption. In this work, the EISCAP modified with TMV particles (as bi-enzyme nanocarriers) was applied to mimic an **XOR** enzyme logic gate.

The **XOR** gate is one of the important elements of biocomputing systems: it provides a true output (**1**) if only one of the inputs is true. If both input signals are false (**0**) or both are true (**1**), it must remain inactive (output signal **0**). As was noted in [86], the enzyme-based **XOR** gate is difficult to realize, because it requires enzymatic reactions that are able to produce approximately equal signals of opposite direction (net output signal should be **0**), when both input signals simultaneously appear. The enzyme/substrate systems used in this work (i.e., urease/urea and penicillinase/penicillin) represented typical examples of such enzymatic reactions producing pH changes and consequently EISCAP signals in opposite direction (see Figure 4).

**Figure 5:**
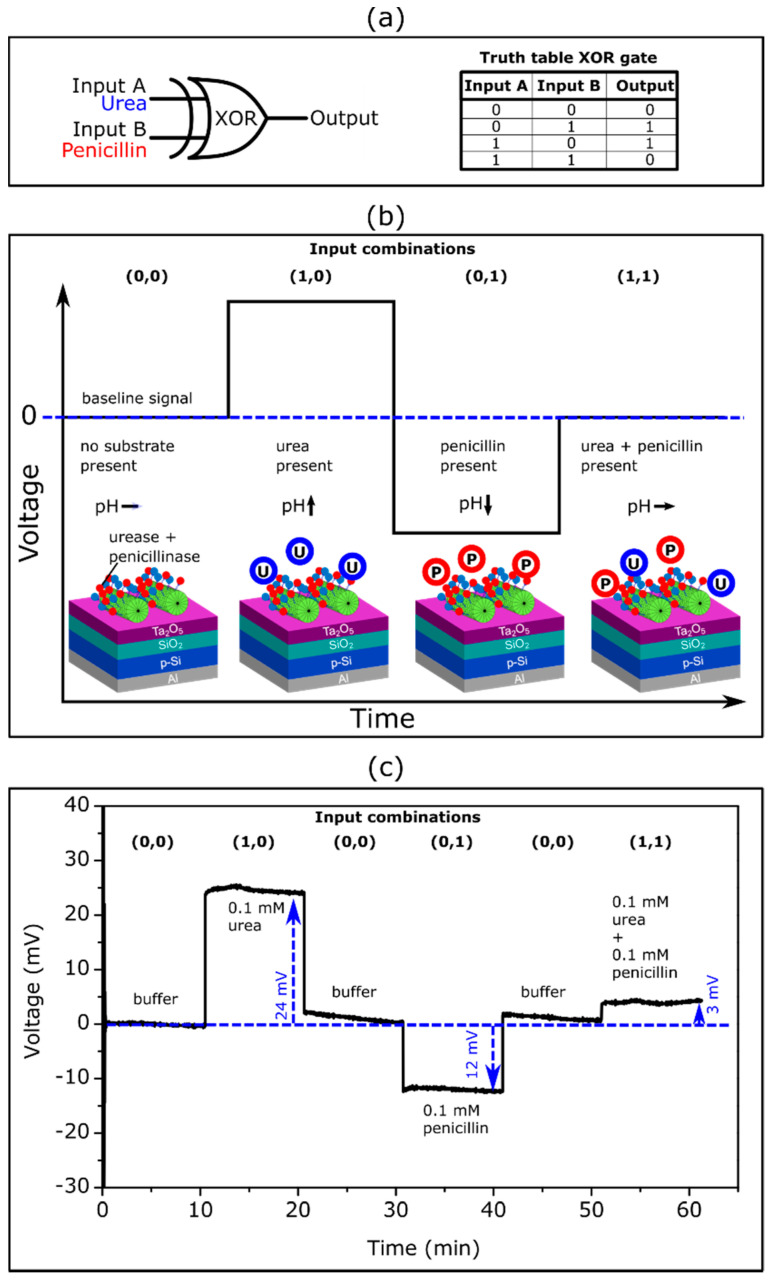
(**a**) Schematic symbol and truth table of an **XOR** gate with urea and penicillin as input signals. (**b**) Expected ConCap response for different input combinations. (**c**) Experimental ConCap curve of the TMV-assisted bi-enzyme EISCAP biosensor recorded in buffer (input combination (**0**,**0**)), in 0.1 mM urea or penicillin solution (input combination (**1**,**0**) or (**0**,**1**)) and in a mixture of 0.1 mM urea and 0.1 mM penicillin solution (input combination (**1**,**1**)). U: urea, P: penicillin.

Figure 5a shows the schematic symbol and truth table for the enzyme-based **XOR** gate with urea as input A and penicillin as input B. The expected ConCap signal of the TMV-assisted bi-enzyme EISCAP biosensor for different input combinations is illustrated in Figure 5b.

The presence of the respective analyte in the solution corresponded to the input signal **1**, while the absence of an analyte was considered as the input signal **0**. When no analyte was present in the solution (input combination (**0**,**0**)), the resulting output signal became **0**, because no local pH change occurred. If one of the analytes (urea or penicillin) was present (input combination (**1**,**0**) or (**0**,**1**)), the output signal became **1** due to the enzymatically induced pH change. The presence of both analytes led to an output signal **0**, as both enzymatic reactions occurred simultaneously with opposing pH shifts which compensated each other.

Figure 5c represents an experimental ConCap logic signal of the TMV-assisted bi-enzyme EISCAP biosensor corresponding to various input combinations: The measurement was started in the buffer solution in the absence of the analyte (input combination (**0**,**0**)), followed by measurement in a 0.1 mM urea (input combination (**1**,**0**)) and 0.1 mM penicillin solution (input combination (**0**,**1**)). To return the local pH to its initial value, after each measurement in the particular analyte solution, the biosensor was exposed to the buffer solution and the baseline signal was recorded again. Finally, the ConCap signal was recorded in the solution containing both 0.1 mM urea and 0.1 mM penicillin (input combination (**1**,**1**)). Relatively large signal shifts of opposite direction were observed when only one of the analytes was present in the solution (24 mV at 0.1 mM urea; 12 mV at 0.1 mM penicillin). In contrast, a very small signal shift of 3 mV was detected if both analytes were present in the solution: two simultaneously occurring enzymatic reactions produced opposing pH changes, resulting in a nearly negligible sensor output signal.

The described proof-of-principle experiment demonstrated the successful application of the TMV-assisted bi-enzyme EISCAPs for the development of **XOR** enzyme logic gates. Future work will be directed to minimize the output signal (should be ideally **0**) of the EISCAP in the presence of both analytes via the optimization of the ratio of urease/penicillinase activities and/or urea/penicillin concentrations in the solution.

## 4. Conclusions

The appropriate enzyme immobilization strategy is a key factor for the design of enzyme-based biosensors. Plant virus-like particles offer the possibility of extremely dense and precise immobilization of enzymes, due to uniform 3D structures and a high density of surface binding sites. In this study, TMV particles were utilized for the co-immobilization of penicillinase and urease onto the Ta_2_O_5_-gate surface of an EISCAP sensor for the serial detection of penicillin and urea. These two enzyme/substrate systems (i.e., penicillinase/penicillin and urease/urea) allowed the performance of typical model experiments with opposing pH shifts on the sensor surface: (a) generating hydrogen ions (penicillinase/penicillin with associated pH decrease) or (b) consuming hydrogen ions (urease/urea with associated pH increase). The sensitive characteristics of TMV-assisted bi-enzyme EISCAPs were characterized in the ConCap mode and compared with those of single-enzyme EISCAPs.

The single-enzyme biosensors offered a high penicillin (95–98 mV/dec) and urea (49–55 mV/dec) sensitivity and remarkable reproducibility. These notable sensor properties were retained even when both enzymes were co-immobilized on the TMV particles, resulting in a penicillin and urea sensitivity of 85 and 54 mV/dec, respectively. The successful application of the TMV-assisted biosensor for designing an **XOR** enzymatic gate further highlighted the potential of the presented sensor arrangement.

The results achieved in this study demonstrated the great prospects of TMV particles as enzyme nanocarriers in constructing EISCAP biosensors for multi-analyte detection. In future studies, TMV-assisted multi-enzyme EISCAPs could be extended to other enzymes. Furthermore, TMV-assisted EISCAPs could be employed for the characterization and application of two- or multi-step enzymatic cascades. Due to the uniform 3D structure of the TMV particles, the enzymes are geometrically close to each other, whereby enzymatic product/substrate diffusion can optimally take place, and the influence of interfering substances could be reduced. This may include not only arrangements with blends of distinct enzymes immobilized on the TMV nanoscaffolds but could also be tailored to enzyme systems collaborating between separate particles. If installed on TMV carriers with length-defined, selectively addressable longitudinal subdomains, such sensors might even provide fundamental insights into spacing-dependent interactions of enzyme groups and, thus, ease the design of high-efficiency artificial biocatalytic systems [87].

## Data Availability

The data presented in this study are available on request from the corresponding author.
